# Molecular Detection of *Brucella* Species Causing Abortion Outbreaks in Ruminant Livestock in Tunisia

**DOI:** 10.1155/ijm/9941176

**Published:** 2025-10-14

**Authors:** Ibtihel Ben Abdallah, Kaouther Guesmi, Awatef Béjaoui, Sana Kalthoum, Amel Arfaoui, Haikel Kessa, Sabeur Hadhiri, Zakia Issaoui, Boubaker Ben Smida, Karima Jouini, Mohamed Bidhani, Aymen Toumi, Chédia Seghaier, Mohamed Naceur Baccar, Abderrazak Maaroufi

**Affiliations:** ^1^Group of Bacteriology and Biotechnology Development, Laboratory of Epidemiology and Veterinary Microbiology, Institut Pasteur de Tunis, University of Tunis El Manar (UTM), Tunis, Tunisia; ^2^Centre National de Veille Zoosanitaire, Ministry of Agriculture, Tunis, Tunisia; ^3^Commissariat Regional au Développement Agricole CRDA, Sousse, Tunisia; ^4^Veterinary Practitioner, Nabeul, Tunisia; ^5^Commissariat Regional au Développement Agricole CRDA, Tataouine, Tunisia; ^6^Veterinary Practitioner, Tozeur, Tunisia; ^7^Office des Terres Domaniales, Tunis, Tunisia

**Keywords:** abortion, *B. abortus*, *B. melitensis*, *Brucella* spp., qPCR testing, ruminant herds, Tunisia, zoonosis

## Abstract

Brucellosis is an endemic zoonotic disease in Africa and Tunisia, severely affecting both human and animal health, particularly ruminants. In livestock, brucellosis causes reproductive failure, including abortions, leading to substantial economic losses. Despite surveillance and vaccination efforts in Tunisia, brucellosis remains widespread. This study is aimed at assessing the presence of *Brucella* infection in aborted animals (sheep, goats, and cattle) using an *IS711*-based real-time PCR assay, determining the circulating species (*Brucella melitensis* and *Brucella abortus*) by differential qPCR, and identifying the most suitable sample type for detection between 2020 and 2022. Samples including vaginal swabs, blood, placenta, and fetal organs (liver, spleen, stomach, and cotyledons) were collected from farms selected based on abortion reports from farm owners. A total of 272 samples were analyzed, of which 24.26% tested positive for *Brucella* spp., with 25.71% in sheep, 13.33% in goats, and 8.33% in cattle. The detection rate of *Brucella* spp. was estimated at 24% (60/250) in aborted females and 27.27% (6/22) in aborted fetal materials. Vaginal swabs exhibited a notable positivity rate of 31.13%, 28.57% in organs and 5.71% in blood. *B. melitensis* and *B. abortus* were detected in 46.96% and 19.69% of positive samples, respectively. Interestingly, one sample showed a coinfection with both species; however, neither *B. abortus* nor *B. melitensis* was detected in 21 positive samples. This study highlights the presence of *Brucella* spp. in aborted ruminants and the circulation of *B. melitensis* and *B. abortus*, underlining the importance of molecular tools for the reliable diagnosis of brucellosis, and their usefulness in mitigating the spread of this infection on farms by applying appropriate control measures.

## 1. Introduction

Brucellosis is a zoonotic disease caused by Gram-negative facultative intracellular bacteria of the genus *Brucella* that affects livestock, wildlife, and humans [1, 2]. Recognizing the significant impact of this disease, the World Organization for Animal Health (WOAH), the Food and Agriculture Organization (FAO), and the World Health Organization (WHO) have identified brucellosis as a significant global zoonotic disease [3, 4]. While brucellosis has been successfully controlled in many developed countries through effective prevention and eradication measures, it is still endemic in developing countries, particularly in Africa, the Middle East, Central Asia, Latin America, and various Mediterranean countries [3, 5]. Human contamination is often associated with animal infection, with approximately 2.1 million new human cases reported annually [6].

In Tunisia, Brucellosis is endemic and listed as a notifiable disease under national regulations. The incidence of human brucellosis in Tunisia has shown an exponential increase, rising from 2.9 cases per 100,000 inhabitants in 2008 to 3.9 cases per 100,000 in 2015 and reaching 9.8 cases per 100,000 in 2017 [7]. Several studies conducted in Tunisia indicate that over 90% of human cases are linked to raw milk consumption, 77% of neurobrucellosis cases to foodborne contamination, and 90.6% of patients report contact with infected animals [8–10]. This persistence is largely attributed to its presence in livestock. Surveillance data over a 14-year period (2005–2018) revealed that the seroprevalence of infected ruminant herds ranged from 0% to 70% of the flocks. Bovine brucellosis is primarily concentrated in the northern and southeastern regions, while small ruminant infections are widespread across most districts [11].

To date, more than 12 different species have been described within the genus *Brucella* [12]. The most virulent species is *Brucella melitensis*, which mainly affects sheep and goats, followed by *Brucella abortus* in cattle and *Brucella suis* in pigs [13, 14]. These species cause significant economic losses in the livestock sector, particularly due to abortion and infertility in females, resulting in reduced milk and meat production [15–17]. In humans, all species but *B. suis* bv. 2 are highly zoonotic, causing a serious and debilitating illness that requires prolonged combined antibiotic therapy. If left untreated, it can lead to severe sequels [18, 19].

The diagnosis of brucellosis traditionally relies on microbiological and serological methods. However, culture methods are time-consuming and require specialized laboratory facilities, and serological tests can lack sensitivity and specificity due to cross-reactivity. To address these issues, molecular techniques such as real-time PCR have been recommended due to their high sensitivity and reliability compared to serological methods [20–22].

Currently, the available data on brucellosis infection and its contribution to abortions in ruminants in Tunisia are fragmented and primarily based on serological methods [8, 11, 23, 24]. However, there have been a few studies that have employed highly sensitive molecular tools [7, 13, 25, 26]. A previous study has employed qPCR to investigate the involvement of brucellosis in bovine abortions, uniquely focusing on the governorate of Sfax in central-eastern Tunisia from 2010 to 2012 [25]. Despite previous studies indicating the presence of brucellosis among aborted ruminants, the actual contribution of this disease to abortion rates on domestic farms remains largely unknown in Tunisia.

Given the health and economic importance of this disease, comprehensive research studies are essential for effective control measures. This study is aimed at identifying *Brucella* species in samples collected from aborted ruminants in various Tunisian locations, between 2020 and 2022. The findings of this research may be very helpful in preventing and controlling the spread of this infection.

## 2. Materials and Methods

### 2.1. Clinical Cases of Abortion in Ruminants

As part of passive surveillance of infectious abortions in ruminants, the Centre National de Veille Zoosanitaire received alerts regarding abortion cases in cattle and small ruminants between February 2020 and November 2022. This management system relies on livestock owners voluntarily reporting cases to veterinary services, enabling veterinarians to conduct on-site investigations and collect samples promptly. Timely reporting is essential to identify the cause, prevent disease transmission, and improve herd health. In most cases, sampling was performed within 24–48 h postabortion; however, in some instances, it occurred more than 1 week after the event. Samples were collected and referred to the Epidemiology and Veterinary Microbiology Laboratory (Institut Pasteur de Tunis) for molecular diagnosis.

**Figure 1:**
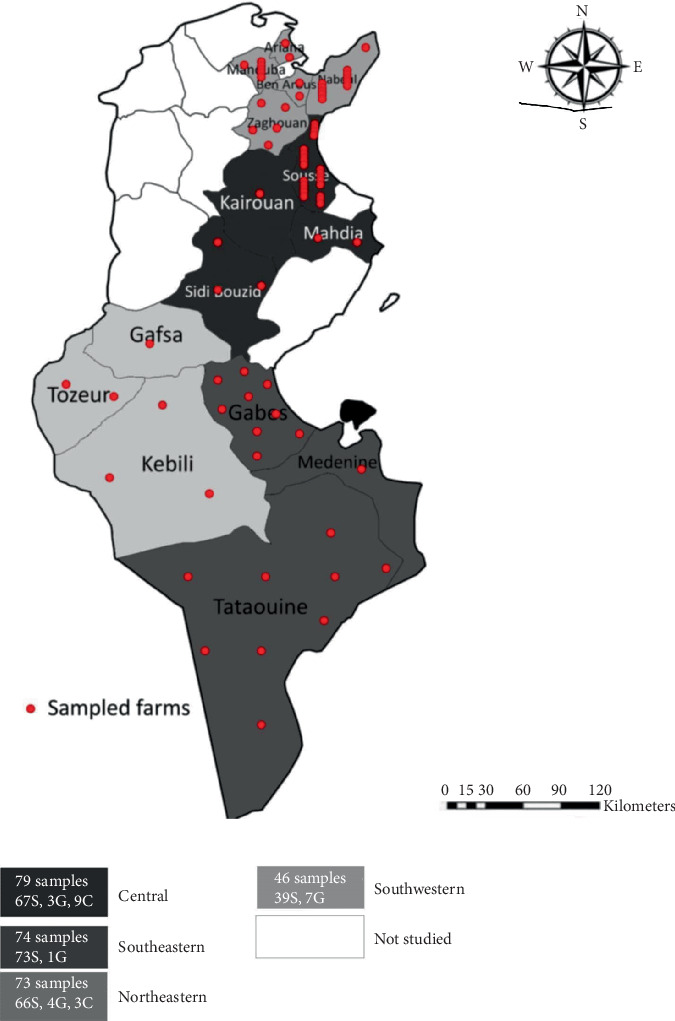
Map of Tunisia showing the geographical distribution of ruminant farms with reported abortion cases, the number of collected samples, and the animal species in four regions over a 3-year period (2020–2022). S: sheep, G: goats, C: cattle.

A total of 79 mixed farms (sheep, goats, and cattle) were included in this study, selected based on reported abortion problems and subsequent veterinary intervention requests. Although the selection was not random, efforts were made to ensure a broad geographic distribution, covering 33 sectors across 15 governorates: five governorates in the northeastern region (Ariana, Manouba, Ben Arous, Zaghouan, and Nabeul), four in the central region (Sousse, Mehdia, Sidi Bouzid, and Kairouan), three in the southeastern region (Gabes, Medenine, and Tataouine), and three in the southwestern region (Gafsa, Tozeur, and Kebili) (Figure 1).

### 2.2. Sample Collection and Processing

For each clinical case, one or more samples (blood, vaginal swabs, placenta, and fetus organs) were taken. A total of 272 samples (100 in 2020, 90 in 2021, and 82 in 2022) were obtained, including 167 vaginal swabs (159 from sheep, 3 from cattle, and 5 from goats), 35 organs (32 from sheep, 1 from cattle, and 2 from goats), and 70 blood samples (55 from sheep, 7 from cattle, and 8 from goats) that were referred to the laboratory for molecular analysis (Table 1). Regarding the analyzed organ types, placentas were collected from 13 cases of aborted females (11 from sheep and 2 from cattle). The other organ types were obtained from 22 aborted fetuses and included 20 from sheep (4 spleens, 4 stomachs, 4 cotyledons, and 8 livers) and 2 from goats (2 livers) (Table 1).

Out of the 272 samples, a collection of 35 blood and swab samples taken simultaneously from the same animals (33 from sheep and 2 from cattle) to determine the most effective sample type for *Brucella* detection using qPCR.

Each sample was individually placed in a sterile bag and transported to the laboratory in a cool box to maintain refrigeration throughout transit. A single positive animal within a flock was sufficient to classify the entire flock as positive.

#### 2.2.1. Biosafety Procedures

To prevent the contamination of personnel and the environment with zoonotic pathogens, high biosecurity measures, protective laboratory clothing, and waste decontamination procedures were applied. All samples were processed under biosafety level two-plus (BSL2+) laboratory, and DNA extraction was conducted in a Class II Type A2 Biosafety Cabinet.

### 2.3. DNA Extraction

The collected vaginal swabs were resuspended in 900 *μ*L of sterile 1× PBS (phosphate-buffered saline), and 500 *μ*L of the suspension was centrifuged for 10 min at 10,000 × g. The obtained pellet was diluted in 100 *μ*L of sterile 1× PBS and used for the extraction of bacterial DNA.

Regarding the organs, the samples were finely minced with a sterile scalpel in an aseptic environment. A total of 30 mg was then used for DNA extraction. For blood samples, a volume of 100 *μ*L was used, and due to the presence of inhibitory substances in the blood, a 1:10 dilution (with sterile double-distilled water) was additionally prepared for DNA extraction.

The DNA extraction was conducted in accordance with the manufacturer's instructions for the QIAamp DNA Mini Kit (QIAGEN, Hilden, Germany). A volume of 100 *μ*L of pathogen-free fetal bovine serum (FBS) (Gibco-BRL, Paisley, United Kingdom) was used as a negative control in the extraction step. The purified DNAs were eluted in 100 *μ*L of AE buffer (QIAGEN, Hilden, Germany) and stored at −20°C until analysis.

### 2.4. qPCR Testing

The TaqMan RT-PCR Bru Multi Assay was performed to detect *Brucella* spp. DNA by amplifying a 121 bp fragment of the *IS711* insertion element gene as previously described [13, 27]. Each 25 *μ*L reaction contained 6.25 *μ*L of TaqMan Environmental Master Mix (Life Technologies, Brant, France), 0.75 *μ*L of each primer (10 *μ*M), 0.25 *μ*L of probe (10 *μ*M), and 8.5 *μ*L of nuclease-free water. Positive control containing *B. abortus* DNA (1 ng/*μ*L) and two negative controls (one for extraction [FBS] and another for amplification [H_2_O/nuclease-free]) were included in each reaction, along with an internal KoMa plasmid DNA control [28]. Each DNA sample was tested in duplicate. The amplification was conducted in a real-time thermocycler, Bio-Rad CFX96 (Bio-Rad, Singapore), and the program consisted of an initial phase at 95°C for 600 s, succeeded by 40 cycles consisting of 15 s at 95°C and 60 s at 60°C. The data analysis was performed using the Bio-Rad CFX Maestro Software. Samples showing cycle threshold (Ct) values of 37 or less were considered positive.

Positive samples were further analyzed by RT-PCR Bru Diff Assay to differentiate *B. abortus* and *B. melitensis*, following the same conditions [13, 27]. All primers and probes used in this study (MOLBIOL, Berlin, Germany) were previously described [13].

## 3. Results

### 3.1. Frequency of *Brucella* spp. in Sheep, Goats, and Cattle

Out of the 272 analyzed samples for the presence of *Brucella*, 66 (24.26%) were found to be positive for qPCR, with a Ct value below 37. The negative controls were confirmed to be valid, with no signal detected. The positive control was also validated in all reactions. The qPCR results indicated an infection rate of 25.71% (63/245) in sheep, followed by 13.33% (2/15) in goats, and 8.33% (1/12) in cattle (Table 2).

The overall detection rate of *Brucella* spp. was estimated at 24% (60/250) in aborted females and 27.27% (6/22) in aborted fetal materials. Among the samples from aborted females, qPCR positivity was highest in vaginal swabs (31.13%, 52/167), followed by placental samples (30.77%, 4/13) and blood samples (5.71%, 4/70). Regarding aborted fetal materials, the spleen had the highest positivity rate (75%, 3/4), followed by the stomach (25%, 1/4) and the liver (20%, 2/10), while none of the cotyledon samples was PCR positive (Table 3).

Furthermore, out of the 35 collections of 35 blood and vaginal swab samples taken simultaneously from the same aborted females, nine (27.27%) tested positive for *Brucella*. Of these, eight positive cases (88.88%) were detected only in vaginal swabs, while the one *Brucella*-positive blood sample (11.11%) was also found to be positive in a vaginal swab sample (Table 4).

Out of the 66 *Brucella*-positive cases, 35 were detected in 2020 (34 in sheep and 1 in goat), 16 in 2021 (15 in sheep and 1 in cattle), and 14 in 2022 (13 in sheep and 1 in goat) (Table 5).

### 3.2. Frequency of *B. melitensis* and *B. abortus*

A qPCR analysis was conducted on all positive samples to identify the present *Brucella* species [29, 30]. Of the 66 positive samples, 46.96% (31/66) were identified as *B. melitensis*, while 19.69% (13/66) were assigned as *B. abortus*. Additionally, one sample (1/66; 1.51%) showed a coinfection with both *B. abortus* and *B. melitensis*.

**Figure 2:**
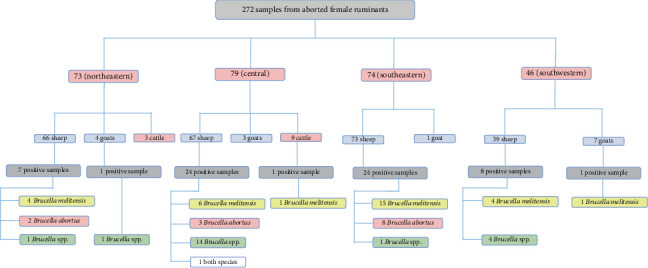
Distribution of *B. abortus* and *B. melitensis* in samples according to four regions of Tunisia (southeastern, central, northeastern, and southwestern) and animal species (sheep, goats, and cattle) over a 3-year period (2020–2022).

A total of 21 (31.81%) of the *Brucella* spp. positive samples were not identified neither as *B. abortus* or *B. melitensis*. The highest positive rates for *B. melitensis* and *B. abortus* were observed in sheep with 46.03% and 20.63%, respectively. In goats, one case of *B. melitensis* and one case of *Brucella* spp. were identified, while in cattle, only one case of *B. melitensis* was observed (Figure 2).

## 4. Discussion

The present study offers updated data on the presence of *Brucella* spp. in aborted female ruminants, including sheep, goats, and cattle during the period between 2020 and 2022, using sensitive qPCR to detect and determine the circulating *Brucella* species and identify the most effective sample types for detecting *Brucella*. The use of molecular tools in diagnostics (PCR and RT-PCR) has addressed limitations associated with classical diagnostic methods such as prolonged processing times and biohazard risks [20, 21, 31]. The choice of qPCR based on the *IS711* gene has been shown as a sensitive and specific assay in comparison to other qPCR tests targeting the *bcsp31* and *per* genes as well as culture and serological tests [29, 31–33]. Indeed, the *IS711* insertion sequence has been demonstrated to be a highly conserved element within the *Brucella* genus, which is present in multiple copies offering low detection limits up to 10 fg per reaction [13, 30].

Our study revealed the presence of *Brucella* DNA in 24.26% (66/272) of samples, underlining the extent of this infection in herds. This is in accordance with other studies confirming the circulation of *Brucella* in ruminant herds in Tunisia and its role as a significant cause of abortion, using qPCR. Indeed, it was reported by Barkallah et al., a notable *Brucella* positivity rate of 31.3% in bovine abortion cases in the Sfax region, in 2012 [25]. Furthermore, Barkallah and collaborators reported, in 2017, a *Brucella* positivity rate of 17.19% in sheep and cattle using qPCR [26].

Brucellosis prevention in Tunisia relies mainly on vaccination. Since 1975, a national program has targeted young dairy cattle (4–7 months) with the B19 vaccine, and in 1991, a similar program was launched for small ruminants using the Rev 1 vaccine for young females (from 3 months) and adults, with annual boosters. However, despite these long-standing vaccination efforts, brucellosis remains endemic [11]. According to our results, the highest number of positive samples was recorded in 2020 (35%), followed by 17.77% in 2021 and 18.29% in 2022. Despite this decreasing trend, the persistence of infection remains evident. This persistent issue emphasizes the need for improved control measures, including more effective vaccination campaigns, better herd management practices, and enhanced surveillance.

On the other hand, when compared to published studies from different countries, the observed percentage in the present study (24.26%) is lower than those reported in Iran (30%) [34], Iraq (32.16%) [35], Kenya (33.3%) [36], and Southeast Europe (37%) [37]. However, it is higher than those reported in Turkey (13, 92%) [31], Colombia (9.5%) [38], and Greece (14.77%) [39].

When comparing *Brucella* positivity rates based on animal species in our study, the infection rates in aborted animals were 25.71% (63/245) for sheep, 13.33% (2/15) for goats, and 8.33% (1/12) for cattle. Barkallah and collaborators have reported 31.3% in cattle in 2014 and 21.49% in cattle and 11.58% in sheep in 2017 in the Sfax region [25, 26]. Based on qPCR studies, positivity rates of 35.45% in sheep and 23.8% in goats in Iraq [34], 6.8% in sheep and 12.5% in goats in Iran [40], and 20.9% in cattle and 10.8% in sheep in Southern Cameroon [41] have been reported. These results clearly illustrate that positivity rates vary depending on the animal species, region, and study period. The detection of *Brucella* spp. in aborted females is estimated at 24% (60/250) and at 27.27% (6/22) in aborted materials. To further understand the distribution of *Brucella* spp. across different sample types and to identify the most effective samples for *Brucella* detection using qPCR, our study analyzed various specimens such as vaginal swabs, placenta, and blood from aborted female ruminants, as well as different fetal organs (including the liver, stomach, cotyledons, and spleen).

In aborted females, *Brucella* spp. DNA was detected most frequently in vaginal swabs, which had an infection rate of 31.13% (52/167), followed by placenta at 30.76% (4/13) and blood at 5.71% (4/70). These results are consistent with our findings from the collection of 35 blood and vaginal swab samples taken simultaneously from the same animals. Of these 35 samples, nine were positive (27.27%). A significant rate of 88.88% (8/9) was observed only in the vaginal swab samples, while the one *Brucella*-positive blood sample (11.11%) (1/9) was also found to be positive in a vaginal swab sample. The lower detection rate in blood may be attributed to the timing of sample collection, as some samples were obtained more than 1 week after abortion events due to the passive nature of surveillance and logistical constraints. This delay likely reduced the likelihood of detecting *Brucella* DNA circulating in the bloodstream.

Moreover, variability in positivity rates was observed across different organ types. The spleen exhibited an infection rate of 75% (3/4), followed by the stomach at 25% (1/4), and the liver at 20% (2/10). These results highlight the importance of selecting the appropriate sample type for detecting *Brucella*. They confirm the high sensitivity of the qPCR method, particularly in vaginal swabs, and suggest that certain organs, notably the spleen and placenta, can increase the probability of detecting *Brucella* in aborted female ruminants. Furthermore, a genital swab taken after abortion or parturition in goats, sheep, or cows appears to be a highly effective biological specimen for the detection and recovery of viable *Brucella* cells [42, 43].

Among the positive samples, *B. melitensis* was identified in 46.96%, *B. abortus* in 19.69%, and one sample (1.51%) demonstrated coinfection with both *B. abortus* and *B. melitensis*. Notably, 31.81% of the *Brucella*-positive samples could not be classified as either *B. melitensis* or *B. abortus*. Both species were most prevalent in sheep, accounting for 46.03% and 20.63%, respectively. One case of *B. melitensis* was also detected in goat and cattle.

These results confirm the circulation of *B. melitensis* and *B. abortus* in ruminant livestock, which could be explained by several factors, including inadequate disposal of aborted materials, lack of cooperation between policymakers and health professionals, and insufficient government compensation for infected animals, which are major obstacles to disease control. Furthermore, hesitancy toward vaccination, uncontrolled animal movements, and the absence of appropriate diagnostic methods further aggravate the situation. Control strategies such as “test-and-slaughter” and milk pasteurization, which have proven effective in developed countries, may not be suitable for developing countries including Tunisia due to resource limitations. These results thus highlight the need for enhanced national and international collaboration, as well as better training for veterinary and medical personnel, to effectively combat brucellosis in these regions [44].

While *B. melitensis* has been linked to infections in sheep and goats, *B. abortus* is typically associated with cattle [45]. However, this host specificity has largely changed, and both species could be found in small ruminants and cattle. Indeed, *B. abortus* in sheep has been reported in Egypt [46], Pakistan [47], Southern Cameroon [41], and Iran [34, 40]. On the other hand, *B. melitensis* in cattle has been reported in Southern Cameroon [41], Tanzania [48], and Pakistan [49], which corroborates with our findings.

Coinfection with both *B. abortus* and *B. melitensis* was detected in sheep (1/66) from a mixed farm. This finding is not unexpected, since several previous studies have reported similar results [21, 40, 41, 46, 50]. These studies highlighted that the cohabitation of different ruminant species within the same farms or shared grazing areas facilitates the transmission of multiple *Brucella* species. The transmission is often facilitated by exposure to secretions from infected cows, particularly under mixed farming conditions. In Tunisia, mixed farming systems are common and occupy 75%–85% of the agricultural land [51]. The practice of herding multiple ruminant species together, which is a traditional activity among sheep breeders in Tunisia, significantly increases the risk of brucellosis transmission between species [21].

## 5. Conclusions

The present study revealed a relatively high occurrence of brucellosis (24.26%) in aborted female ruminants, as detected by qPCR, indicating a widespread presence of the disease in the study region of Tunisia. The qPCR method demonstrated high sensitivity, particularly when applied to vaginal swabs and fetal organs, such as the spleen and placenta. Both species *B. melitensis* and *B. abortus* were detected, posing a potential risk to human health. However, as the study was conducted in the context of diagnosing passive abortion, the results may not accurately reflect the true prevalence of the disease in livestock. The study confirmed the need to strengthen vaccination programs for ruminants and to raise public awareness of brucellosis.

## Figures and Tables

**Table 1:** Distribution of sample types collected from sheep, goats, and cattle.

**Sample type**	**Total (** **n** **)**	**Sheep (** **n** **)**	**Goats (** **n** **)**	**Cattle (** **n** **)**
Vaginal swabs	167	159	5	3
Blood	70	55	8	7
Organs	35	32	2	1
• Placenta	13	11	0	2
• Fetal spleen	4	4	0	0
• Fetal stomach	4	4	0	0
• Fetal liver	10	8	2	0
• Fetal cotyledon	4	4	0	0
Total (*n*)	272	245	15	12

Abbreviation: *n*, number.

**Table 2:** Percentage of *Brucella* spp. in aborted domestic ruminants in Tunisia over a 3-year period (2020–2022).

**Animal species**	**Total number**	**No. of positive**	**Percentage (%)**
Sheep	245	63	25.71
Goats	15	2	13.33
Cattle	12	1	8.33
Total	272	66	24.26

**Table 3:** Detection of *Brucella* spp. in different types of samples.

**Origin**	**Samples**	**Total number**	**No. of positive**	**Percentage (%)**
Aborted female	Vaginal swabs	167	52	31.13
Ruminants	Blood	70	4	5.71
	Placenta	13	4	30.76
Aborted	Liver	10	2	20
Fetal	Stomach	4	1	25
Materials	Spleen	4	3	75
	Cotyledon	4	0	0

**Table 4:** Results of qPCR on 35 blood and vaginal swab samples collected simultaneously from the same aborted females.

**Animal species**	**No. of tested animals**	**No. of positive**	**qPCR result**		
			**Vaginal swab samples**	**Blood samples**	**Both samples**
Sheep	33	9/33 (27.27%)	8/9 (88.88%)	0/9 (0%)	1/9 (11.11%)
Cattle	2	0/2 (0%)	0%	0%	0%

**Table 5:** Data for animal samples tested for *Brucella* spp. in sheep, goats, and cattle in different regions of Tunisia from 2020 to 2022.

**Year**	**Animal species**	**Total number**	**No. of positive**	**Total positive cases**
2020	Sheep	93	34	35 (35%)
	Goats	7	1	

2021	Sheep	79	15	16 (17.77%)
	Goats	5	0	
	Cattle	6	1	

2022	Sheep	73	14	15 (18.29%)
	Goats	3	1	
	Cattle	6	0	

## Data Availability

The data that support the findings of this study are available from the corresponding author upon reasonable request.

## References

[1] Qureshi K. A., Parvez A., Fahmy N. A. (2023). Brucellosis: Epidemiology, Pathogenesis, Diagnosis and Treatment–A Comprehensive Review. *Annals of Medicine*.

[2] Cloeckaert A., Roop R. M., Scholz H. C., Whatmore A. M., Zygmunt M. S. (2024). Editorial: Pathogenomics of the Genus *Brucella* and Beyond, Volume II. *Frontiers in Microbiology*.

[3] Corbel M. J., Food and Agriculture Organization of the United Nations, World Health Organization, World Organization for Animal Health (2006). *Brucellosis in Humans and Animals*.

[4] Hull N. C., Schumaker B. A. (2018). Comparisons of Brucellosis Between Human and Veterinary Medicine. *Infection Ecology & Epidemiology*.

[5] Buttigieg S. C., Savic S., Cauchi D., Lautier E., Canali M., Aragrande M. (2018). Brucellosis Control in Malta and Serbia: A One Health Evaluation. *Frontiers in Veterinary Science*.

[6] Laine C. G., Johnson V. E., Scott H. M., Arenas-Gamboa A. M. (2023). Global Estimate of Human *Brucellosis* Incidence. *Emerging Infectious Diseases*.

[7] Selmi R., Mamlouk A., Belkahia H. (2024). Serological and Molecular Survey of Brucellosis and Chlamydiosis in Dromedary Camels From Tunisia. *Comparative Immunology, Microbiology and Infectious Diseases*.

[8] Khbou M. K., Htira S., Harabech K., Benzarti M. (2018). First Case-Control Study of Zoonotic *Brucellosis* in Gafsa District, Southwest Tunisia. *One Health*.

[9] Oueslati I., Berriche A., Ammari L. (2016). Epidemiological and Clinical Characteristics of Neurobrucellosis Case Patients in Tunisia. *Médecine et Maladies Infectieuses*.

[10] Koubaa M., Maaloul I., Marrakchi C. (2014). Spinal *Brucellosis* in South of Tunisia: Review of 32 Cases. *The Spine Journal*.

[11] Guesmi K., Kalthoum S., Belhaj Mohamed B., Ben Aicha I., Hajlaoui H., Hrabech K. (2020). Bilan de la Brucellose Animale et Humaine en Tunisie: 2005–2018. *Bulletin Zoosanitaire*.

[12] Daugaliyeva A., Daugaliyeva S., Kydyr N., Peletto S. (2024). Molecular Typing Methods to Characterize *Brucella* spp. From Animals: A Review. *Veterinary World*.

[13] Béjaoui A., Ben Abdallah I., Maaroufi A. (2022). *Brucella* spp. Contamination in Artisanal Unpasteurized Dairy Products: An Emerging Foodborne Threat in Tunisia. *Food*.

[14] Rajendhran J. (2021). Genomic Insights Into *Brucella*. *Infection, Genetics and Evolution*.

[15] Lokamar P. N., Kutwah M. A., Atieli H., Gumo S., Ouma C. (2020). Socio-Economic Impacts of *Brucellosis* on Livestock Production and Reproduction Performance in Koibatek and Marigat Regions, Baringo County, Kenya. *BMC Veterinary Research*.

[16] Shekhar C. (2018). Impact of *Brucellosis* on Health and Economy. *Research Journal of Veterinary Sciences*.

[17] Ibarra M., Campos M., Ibarra C. (2023). Financial Losses Associated With Bovine Brucellosis (*Brucella abortus*) in Carchi-Ecuador. *Open Journal of Animal Sciences*.

[18] Aragón-Aranda B., De Miguel M. J., Lázaro-Antón L. (2020). Development of Attenuated Live Vaccine Candidates Against Swine Brucellosis in a Non-Zoonotic B. *suis* Biovar 2 Background. *Veterinary Research*.

[19] Moriyón I., Blasco J. M., Letesson J. J., De Massis F., Moreno E. (2023). *Brucellosis* and One Health: Inherited and Future Challenges. *Microorganisms*.

[20] Etemadi A., Moniri R., Neubauer H., Dasteh Goli Y., Alamian S. (2019). Laboratory Diagnostic Procedures for Human *Brucellosis*: An Overview of Existing Approaches. *Jundishapur Journal of Microbiology*.

[21] Zare Bidaki M., Allahyari E., Zeinali T., Asgharzadeh M. (2022). Occurrence and Risk Factors of *Brucellosis* Among Domestic Animals: An Artificial Neural Network Approach. *Tropical Animal Health and Production*.

[22] Rahimoon M. M., Mirani A. H., Sahito J. K. (2024). *Brucellosis* and Its Diagnostic Techniques in Animals: A Comprehensive Review. *Journal of Bioresource Management*.

[23] Elandalousi R. B., Ghram A., Maaroufi A., Mnif W. (2015). Séroprévalence des Maladies Abortives Zoonotiques Chez les Ruminants au Nord de la Tunisie. *Research*.

[24] Guesmi K., Kalthoum S., Mamlouk A. (2023). Seroprevalence of Zoonotic Abortive Diseases and Their Associated Risk Factors in Tunisian Sheep. *BMC Veterinary Research*.

[25] Barkallah M., Gharbi Y., Hassena A. B. (2014). Survey of Infectious Etiologies of Bovine Abortion During Mid- to Late Gestation in Dairy Herds. *PLoS One*.

[26] Barkallah M., Gharbi Y., Zormati S. (2017). A Mixed Methods Study of Ruminant Brucellosis in Central-Eastern Tunisia. *Tropical Animal Health and Production*.

[27] Probert W. S., Schrader K. N., Khuong N. Y., Bystrom S. L., Graves M. H. (2004). Real-Time Multiplex PCR Assay for Detection of *Brucella* spp., *B. abortus*, and *B. melitensis*. *Journal of Clinical Microbiology*.

[28] Kirchner S., Krämer K. M., Schulze M. (2010). Pentaplexed Quantitative Real-Time PCR Assay for the Simultaneous Detection and Quantification of Botulinum Neurotoxin-Producing Clostridia in Food and Clinical Samples. *Applied and Environmental Microbiology*.

[29] Becker G. N., Tuon F. F. (2021). Comparative Study of IS711 and Bcsp31-Based Polymerase Chain Reaction (PCR) for the Diagnosis of Human Brucellosis in Whole Blood and Serum Samples. *Journal of Microbiological Methods*.

[30] Aljanazreh B., Shamseye A. A., Abuawad A., Ashhab Y. (2023). Genomic Distribution of the Insertion Sequence *IS711* Reveal a Potential Role in *Brucella* Genome Plasticity and Host Preference. *Infection, Genetics and Evolution*.

[31] Yeni D. K. (2024). Evaluation of Culture and Real Time PCR Methods for the Diagnosis of *Brucellosis*. *Research & Practice in Veterinary & Animal Science*.

[32] Bounaadja L., Albert D., Chénais B. (2009). Real-Time PCR for Identification of *Brucella* spp.: A Comparative Study of *IS711*, *Bcsp31* and *per* Target Genes. *Veterinary Microbiology*.

[33] Zakaria A. M. (2018). Comparative Assessment of Sensitivity and Specificity of Rose Bengal Test and Modified In-House ELISA by Using *IS711* TaqMan Real Time PCR Assay as a Gold Standard for the Diagnosis of Bovine Brucellosis. *Biomedical and Pharmacology Journal*.

[34] Dadar M., Alamian S. (2021). Identification of Main *Brucella* Species Implicated in Ovine and Caprine Abortion Cases by Molecular and Classical Methods. *Archives of Razi Institute*.

[35] Ameen A. M., Abdulaziz N. S., Ghaffar N. M. (2023). Molecular Detection and Associated Risk Factors of *Brucella melitensis* in Aborted Sheep and Goats in Duhok Province, Iraq. *Pathogens*.

[36] Akoko J. M., Pelle R., Lukambagire A. S. (2021). Molecular Epidemiology of *Brucella* Species in Mixed Livestock-Human Ecosystems in Kenya. *Scientific Reports*.

[37] Maksimović Z., Jamaković A., Semren O., Rifatbegović M. (2022). Molecular Detection of *Brucella* spp. in Clinical Samples of Seropositive Ruminants in Bosnia and Herzegovina. *Comparative Immunology, Microbiology and Infectious Diseases*.

[38] Ramírez O. L. H., Santos H. A., Paulino P. G. (2022). Cross-Sectional Study of *Brucella* spp. Using Real-Time PCR From Bovine Whole Blood in Colombia. *Veterinary Research Communications*.

[39] Katsiolis A., Papanikolaou E., Stournara A. (2022). Molecular Detection of *Brucella* spp. in Ruminant Herds in Greece. *Tropical Animal Health and Production*.

[40] Rostami S., Rashidian E., Jaydari A., Rahimi H. (2023). Investigation of the Proportion of *Brucella abortus* and *Brucella* melitensis in Sheep and Goat Milk. *Veterinary Medicine International*.

[41] Mitterran K. N. R., Barberine S. A., Oumarou F., Simo G. (2020). Detection of *Brucella abortus* and *Brucellla melitensis* in Cattle and Sheep from Southern Cameroon.

[42] Freddi L., Djokic V., Petot-Bottin F. (2021). The Use of Flocked Swabs With a Protective Medium Increases the Recovery of Live *Brucella* spp. and DNA Detection. *Microbiology Spectrum*.

[43] Poester F. P., Samartino L. E., Santos R. L. (2013). Pathogenesis and Pathobiology of *Brucellosis* in Livestock. *Revue Scientifique et Technique*.

[44] Hikal A., Wareth G., Khan A. (2023). Brucellosis: Why Is It Eradicated From Domestic Livestock in the United States but Not in the Nile River Basin Countries?. *Journal of Microbiology*.

[45] Fiebig A., Vrentas C. E., Le T. (2021). Quantification of *Brucella abortus* Population Structure in a Natural Host. *Proceedings of the National Academy of Sciences*.

[46] Wareth G., Melzer F., Tomaso H., Roesler U., Neubauer H. (2015). Detection of *Brucella abortus* DNA in Aborted Goats and Sheep in Egypt by Real-Time PCR. *BMC Research Notes*.

[47] Saleem M. Z., Akhtar R., Aslam A. (2019). Evidence of *Brucella abortus* in Non-Preferred Caprine and Ovine Hosts by Real-Time PCR Assay. *Pakistan Journal of Zoology*.

[48] Mengele I. J., Akoko J. M., Shirima G. M. (2024). *Brucella* Species Circulating in Smallholder Dairy Cattle in Tanzania. *Pathogens*.

[49] Ullah I., Naz S., Khattak U. S., Saeed M., Akbar N. U., Rauf S. (2024). Molecular Prevalence, Phylogenetic Analysis, and PCR-Based Detection of *Brucella melitensis* in Humans and Cattle in Southern Khyber Pakhtunkhwa, Pakistan. *Comparative Immunology, Microbiology & Infectious Diseases*.

[50] Akoko J., Pelle R., Schelling E. (2019). Diversity of Brucellosis and Multi-Strain Co-Infection in a Transboundary Livestock Production System Between Tanzania and Kenya. *Open Res Africa*.

[51] Dhehibi B., Fouzai A., Frija A. (2023). Assessing Complementary Synergies for Integrated Crop–Livestock Systems Under Conservation Agriculture in Tunisian Dryland Farming Systems. *Frontiers in Sustainable Food Systems*.

